# One-dimensional proteomic profiling of *Danio rerio* embryo vitellogenin to estimate quantum dot toxicity

**DOI:** 10.1186/s12953-015-0072-7

**Published:** 2015-05-02

**Authors:** Natalia A Petushkova, Galina P Kuznetsova, Olesya V Larina, Yulia S Kisrieva, Natalia F Samenkova, Oxana P Trifonova, Yuliana V Miroshnichenko, Konstantin V Zolotarev, Irina I Karuzina, Olga M Ipatova, Andrey V Lisitsa

**Affiliations:** Orekhovich Institute of Biomedical Chemistry, 119121, Pogodinskaya St. 10, Moscow, Russia; Postgen Tech LLC, 119121, Pogodinskaya St. 10, Moscow, Russia

**Keywords:** *Danio rerio* embryos, Quantum dot, SDS-PAGE, MALDI-TOF mass spectrometry, Vitellogenin cleavage product

## Abstract

**Background:**

Vitellogenin (Vtg) is the major egg yolk protein (YP) in most oviparous species and may be useful as an indicator in ecotoxicological testing at the biochemical level. In this study, we obtained detailed information about the Vtgs of *Danio rerio* embryos by cutting SDS-PAGE gel lanes into thin slices, and analyzing them slice-by-slice with (MALDI-TOF) mass spectrometry.

**Results:**

We conducted three proteomic analyses, comparing embryonic *Danio rerio* Vtg cleavage products after exposure for 48 h to CdSecore/ZnSshell quantum dots (QDs), after exposure to a mixture of the components used for quantum dot synthesis (MCS-QDs), and in untreated embryos. The Vtg mass spectrometric profiles of the QDs-treated embryos differed from those of the unexposed or MCS-QDs-treated embryos.

**Conclusion:**

This study demonstrates the possible utility of Vtg profiling in *D. rerio* embryos as a sensitive diagnostic tool to estimate nanoparticle toxicity.

**Electronic supplementary material:**

The online version of this article (doi:10.1186/s12953-015-0072-7) contains supplementary material, which is available to authorized users.

## Background

The *Danio rerio* embryo is a valuable model organism, widely used in biological and biotechnological research [1,2]. *Danio rerio* is also a recommended test species for various toxicological standard tests, including OECD, ISO, and US EPA Guidelines. Analysis of the *D. rerio* proteome has been used to study the mechanisms of embryo development [3-6], and is also used to study the pathogenesis, prognosis, treatment, and biomarkers of human diseases [7,8].

The main problem encountered with the proteomic analysis of *D. rerio* embryos is the high content of yolk proteins (YPs) derived from vitellogenins (Vtgs). Vtgs are glycophospholipoproteins with an apparent molecular weight of 350–370 kDa, with Ca^2+^ and Zn^2+^ as ligands [9,10]. In the yolk, Vtg is proteolytically cleaved into the heavy (~120 kDa, LvH) and light (~30–35 kDa, LvL) lipovitellin chains, phosvitin (Pv), beta’-component, C-terminal peptide, and various Lv–Pv complexes [11,12]. *Danio rerio* Vtgs are the products of seven different genes and are classified into three types based on their primary amino acid sequences: type I, including Vtg1 and Vtg4–7; type II, containing Vtg2; and type III, containing Vtg3. According to the revised nomenclature of the Vtgs of ostariophysian fishes, the *D. rerio* YPs derived from the multiple fish Vtgs have been reclassified as VtgAo1, VtgAo2, and VtgC, respectively [13,14]. The major *D. rerio* protein is VtgI, which is expressed 100 times more strongly than *Vtg2* and 1000 times more strongly than *Vtg3* [15].

The association between Vtg and oocyte growth is well known. Vtg is synthesized in the livers of female teleost fishes and transported to the oocytes, where it is used as nutrient for their growth and development [11]. The selective regulation of the uptake and processing of multiple Vtgs during oocyte growth and ovarian maturation are probably critical for egg quality and developmental success. The dramatic downregulation of the expression of this nutritional protein might be an important cause of embryonic growth retardation [16]. Because these processes appear to be species specific, their characterization in individual species of interest is necessary to understand its normal reproductive biology and any pathology or dysfunction [17]. In recent years, it has been suggested that the Vtgs are associated with defense reactions, and act as an acute-phase protein *in vivo*, facilitating the elimination of invading pathogens in both female and male fish [18].

A high Vtg content in embryos may hamper the detection of changes occurring in the proteome. However, Vtg and its cleavage products may have utility as indicators of ecotoxicology at the biochemical level [19,20]. Embryos allow the visible effects of treatment with chemicals to be monitored during development. Furthermore, the use of larval and juvenile zebrafish for screening tests in toxicity studies has allowed smaller doses of toxin to be used [21].

Western blotting analyses, enzyme-linked immunosorbent assays (ELISAs), quantitative real-time PCR, and Vtg mRNA determination with DNA hybridization strategies have all been used to evaluate the changes in Vtg levels in fish [15,22,23]. Proteomic approaches allow the direct estimation of protein abundance, posttranslation modification, and protein–protein interactions [24]. Two-dimensional electrophoresis (2DE) combined with mass spectrometry (MS) is a conventional method for studying the *D. rerio* proteome and for identifying differences between samples [4,19,25,26]. For example, Gündel et al. [19] used silver-stained 2DE gels followed by matrix-assisted laser desorption-ionization time-of-flight (MALDI-TOF/TOF) and electrospray ionization-tandem MS (ESI-MS/MS) to identify 47 proteins in *D. rerio* embryos, the bulk of which (93%) were Vtgs, and 90% of all the Vtg derivatives were assigned to Vtg1 (VtgAo1).

However, the 2DE procedure has certain limitations and problems. For instance, it is a labor-intensive method, with relatively low reproducibility, resolution, and separation of very acidic and/or very basic proteins. Alternatively, SDS-PAGE displays high reproducibility and broad resolution, allowing the separation of proteins with apparent MWs of 10–450 kDa on the same gel. A number of bands associated with Vtgs can be distinguished from other proteins on SDS-PAGE gels [12,17,27,28]. After separation, the stained bands corresponding to YPs are excised and the Vtgs in these bands identified [17,29]. During the development of fish embryos, especially the embryos of marine species, some of these protein bands either disappear completely or their intensity decreases, whereas others appear for the first time or their intensities increase [15].

In addition to SDS-PAGE and 2DE, the fractionation of complex samples by centrifugation has also been reported to increase the total number of ovary (membrane and cytosolic) proteins recovered [30]. Data-dependent analysis (also referred to as “shotgun proteomics”) is currently the MS method most frequently used to examine the multiplicity of Vtgs in different fish species [12,28,31].

We previously proposed a one-dimensional proteomic mapping approach based on the cutting of SDS-PAGE gel lanes and the subsequent MALDI-TOF peptide mass fingerprinting (PMF) analysis of each slice, to increase the number of proteins identified [32,33]. We evaluated the application of this approach to the study of the *D. rerio* protein profile and its changes in response to doxorubicin exposure [34]. Here, we used the same strategy to analyze the one-dimensional proteomic profiles of the Vtgs of *D. rerio* embryos in response to exposure to quantum dot (QDs).

Fluorescent semiconductor nanocrystals, or QDs, have become an essential tool in biomedical imaging and diagnostics. QDs consist of a semiconductor core (for example, CdSe or CdTe), and a shell (e.g., ZnS) that improves their optical and electronic properties [35] and reduces core metal leaching. These nanocrystals allow the study of the processes within a cell at the level of individual molecules, with high resolution. However, the toxicity of QDs has not been sufficiently investigated, although it has been shown that the cytotoxicity of some QDs is associated with their constituent cadmium ions [1,36]. An effective way to reduce QD toxicity is to encapsulate them in a layer of polymer of different types. This protects QDs against the action of enzymes and other biological molecules, increases their dispersibility in water, and directs them to biological targets [36]. The purpose of this study was to develop a proteomic approach to determine the toxicity of QDs (CdSe_core_/ZnS_shell_ coated with dihydrolipoic acid [DHLA]) based on the changes they induce in the one-dimensional proteomic profiles of Vtg cleavage products.

## Results

In this study, the profiles of the Vtg cleavage products of *D. rerio* embryos after exposure to QDs for 48 h were analyzed and compared with their profiles after exposure to MCS-QDs. Embryos growing in medium without toxic substances were used as the control.

### QD characterization

A schematic image and the optical properties of the CdSe_core_/ZnS_shell_ QDs are shown in Additional file 1: Figure S1. According to these data, the QDs investigated have a spherical structure (Additional file 1: Figure S1a). The size distribution histogram indicates that the average size of the QDs is around 9.5 nm (Additional file 1: Figure S1b). The absorption spectrum shows a peak at 585 nm (Additional file 1: Figure S1c), and the emission spectrum (**λ**_ex_ = 312 nm) has a maximum at 605 nm, and appears rather broad (Additional file 1: Figure S1d). The QDs used in this study were stable in aqueous solution. The fluorescence quantum yield was ≤ 1%.

### Separation of embryos and embryonic proteins with SDS-PAGE

**Figure 1:**
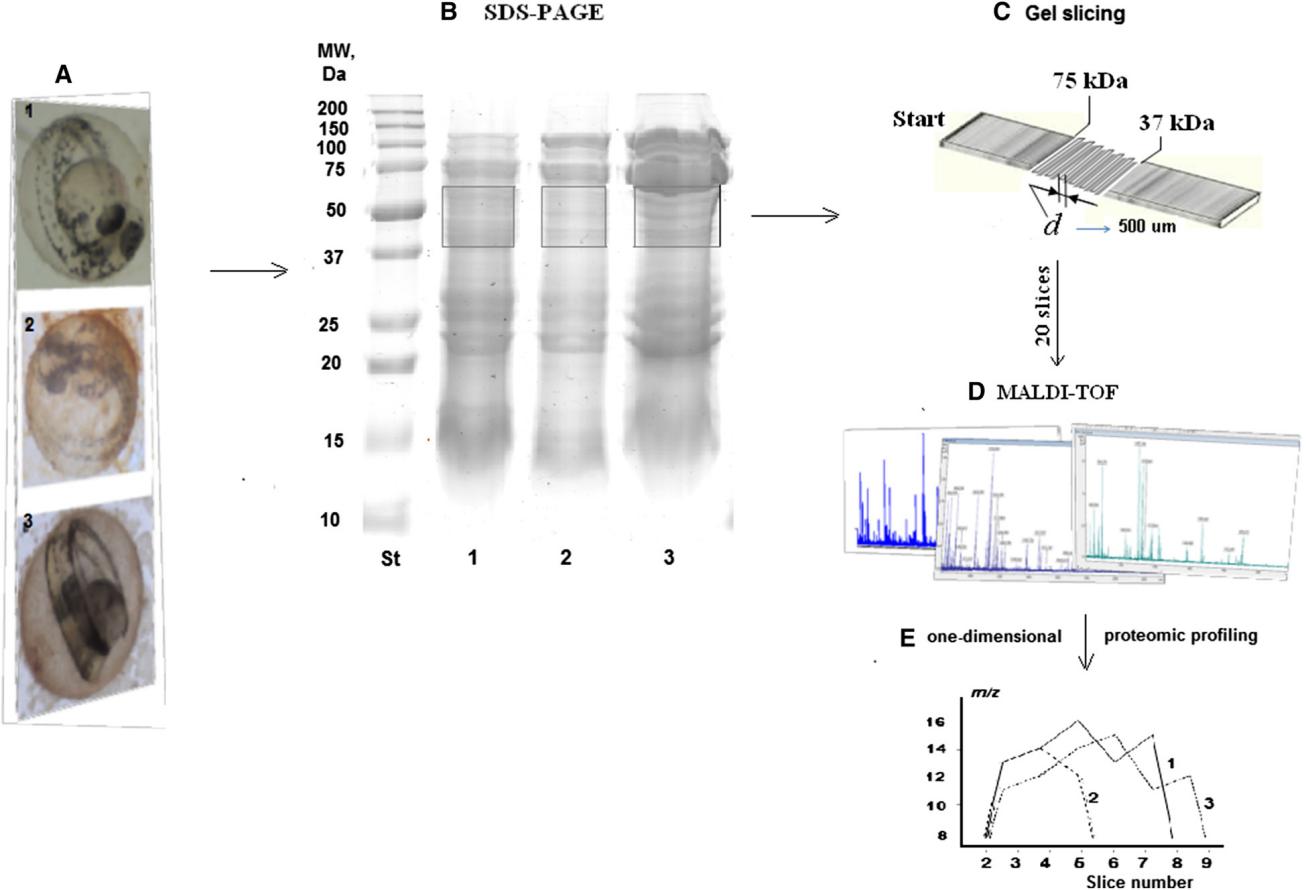
Schematic representation of the strategy used to analyze *Danio rerio* embryo proteins. **(A)** Three *Danio rerio embryo lines* **(B)** SDS-PAGE separation of *D. rerio* embryos proteins. The gel was stained with Coomassie Brilliant Blue to visualize the protein bands. The regions of the gels corresponding to molecular weights of 37–75 kDa, which were excised for Vtg identification, are marked with squares **(C)** SDS-PAGE gel slicing **(D)** One-dimensional proteomic profiles (1) a normally developed 2 days postfertilization (*dpf*) *D. rerio* embryo; (2) normally developed *D. rerio* embryo exposed for 48 h to QD suspension; (3) normally developed 2 *dpf D. rerio* embryo exposed to a mixture of the components used for QD synthesis (0.045 mM Cd^2+^). * DHLA (dihydrolipoic acid).

Figure 1a1 shows the control embryos at the 52 h postfertilization (*hpf*) stage of development, when the embryo is fully formed (tail section is separated, brain, digestive, and excretory systems are formed; there is a functioning heart and blood vessels). Under our experimental conditions, the presence of QDs in the embryo medium did not affect embryonic development throughout the 48 h of treatment (Figure 1a2); nor did MCS-QDs in the medium affect the developing embryos (Figure 1a3).

We used the strategy in which SDS-PAGE gel lanes are sequentially thinly sliced and the proteins therein are identified with slice-by-slice MS (Figure 1b-e). In the first stage, *D. rerio* embryo proteins were separated with SDS-PAGE. Equal amounts of protein (an aliquot of an ultrasonicated homogenate, containing four embryos) from both the unexposed embryos (Figure 1b1) and the embryos exposed to QDs (Figure 1b2) or MCS-QDs (Figure 1b3) were loaded into six separate lanes (two lanes *per* embryonic sample) of two replicate gels for each of the two series of QD exposure assessments. Bio-Rad Precision Plus Protein™ WesternC™ Standards (250, 150, 100, 75, 50, 37, 25, 20, 15, and 10 kDa) were also loaded onto each gel. The regions corresponding to 15, 20–30, and 37–75 kDa showed the most intensive staining (Figure 1b). Depending on the sample, 15–18 separate protein bands were apparent in each single lane after Coomassie Brilliant Blue staining. For instance, in lane 1 (Figure 1b), corresponding to the unexposed embryos, the 37–75 kDa region contained 6–7 bands; exposure to the QDs (lane 2, Figure 1b) or MCS-QDs (lane 3, Figure 1b) negligibly altered the number of protein bands in the selected MW region compared with the control (six protein bands in each case).

### MALDI-TOF identification of Vtg cleavage products in *D. rerio* embryos

To characterize the Vtg cleavage products of whole *D. rerio* embryos, the region corresponding to molecular masses of about 37–75 kDa in each SDS-PAGE gel lane was chosen (Figure 1b). The region selected in each gel lane was cut into a sequential series of thin slices (Figure 1c). In total, 20 slices were obtained *per* lane for each embryo treatment group (the unexposed and the two treated sets of embryos), each measuring 0.5 mm × 0.8 mm × 2 mm. The proteins were identified with PMF after in-gel digestion (Figure 1d). Of the several MALDI-TOF mass spectra obtained for each of the three positions on the MALDI target corresponding to each slice, the best one (i.e., the spectrum with the greatest score for identified protein and the highest percentage of sequence coverage) was selected. The average number of peptide peaks in the PMF spectrum was calculated to be 64 ± 9 for the control embryos, 56 ± 13 for the QD-treated embryos, and 73 ± 14 for the MCS-QD-treated embryos.

Twelve Vtg products, with MWs of 90–180 kDa, were successfully identified in the unexposed *D. rerio* embryos (Table 1). Exposure to QDs or MCS-QDs did not cause any changes in the total number of Vtgs identified (Table 1). Vtgs type II (VtgAo2) Q1MTC4 (MW 179783 Da) and F1R876 (93213 Da) were detected in all the samples, in addition to the Vtg type I (VtgAo1) proteins.

**Table 1:** **Identified vitellogenin (Vtg) cleavage products in the** ***Danio rerio*** **embryos (number of** ***m/z*** **peaks, that match the peptides of the protein are given as Mean ± SD)**

**№**	**Accession** ^*^ **(Swiss-Prot)**	**Protein name**	**Mw, Da**	**Previous homologue name**	**Reclassified vitellogenin homologue**	**Possible Vtg-derived structural conjugates of yolk proteins**	**Number of** ***m/z*** **peaks, that match the peptides of the protein**		
							**Control**	**Quantum dots**	**MCS-QDs**
1	Q1LWN2	Vitellogenin 1	149140	VtgI	VtgAo1	Full Vtg	28 ± 5	24 ± 8	26 ± 6
2	Q1MTC4	Vitellogenin 2	179783	VtgII	VtgAo2	LvH-Pv-LvL-β’	23 ± 4	25 ± 5	25 ± 4
3	F1R876	Vitellogenin 2	93213	VtgII	VtgAo2	Pv-LvL- β’-CT	16 ± 2	19 ± 3	15 ± 1
4	F1R887^**^ deleted	Vitellogenin 4	148750	VtgI	VtgAo1	Full Vtg	28 ± 7	22 ± 8	23 ± 6
5	F1Q7L0	Vitellogenin 4 (Fragment)	149254	VtgI	VtgAo1	Full Vtg	26 ± 5	21 ± 8	23 ± 6
6	E9QFD8	Vitellogenin 4	149258	VtgI	VtgAo1	Full Vtg	25 ± 6	22 ± 8	24 ± 6
7	F1RBA0	Vitellogenin 4	124048	VtgI	VtgAo1	LvH-Pv	22 ± 4	17 ± 6	20 ± 4
8	F1QTW6^**^ deleted	Vitellogenin 5	148776	VtgI	VtgAo1	Full Vtg	22 ± 3	20 ± 5	21 ± 5
9	F1R2S5	Vitellogenin 5 (Fragment)	149280	VtgI	VtgAo1	Full Vtg	22 ± 4	20 ± 5	22 ± 5
10	F1QV15	Vitellogenin 6 (Fragment)	149920	VtgI	VtgAo1	Full Vtg	23 ± 4	21 ± 6	21 ± 6
11	Q1MTC6	Vitellogenin 7	147084	VtgI	VtgAo1	LvH-Pv-LvL	23 ± 4	22 ± 5	22 ± 4
12	F1R2T3	Vitellogenin 7	148896	VtgI	VtgAo1	Full Vtg	21 ± 3	23 ± 5	21 ± 4

The reclassified Vtg gene homologue nomenclature and Vtg-derived structural conjugates are given according to Finn [13]. Full Vtg, LvH-Pv-LvL- β’-CT; LvH, lipovitellin heavy chain; Pv, phosvitin; LvL, lipovitellin light chain; β’, beta’ component; CT, C-terminal coding region. *release 2014_04; **release 2015_02.

**Figure 2:**
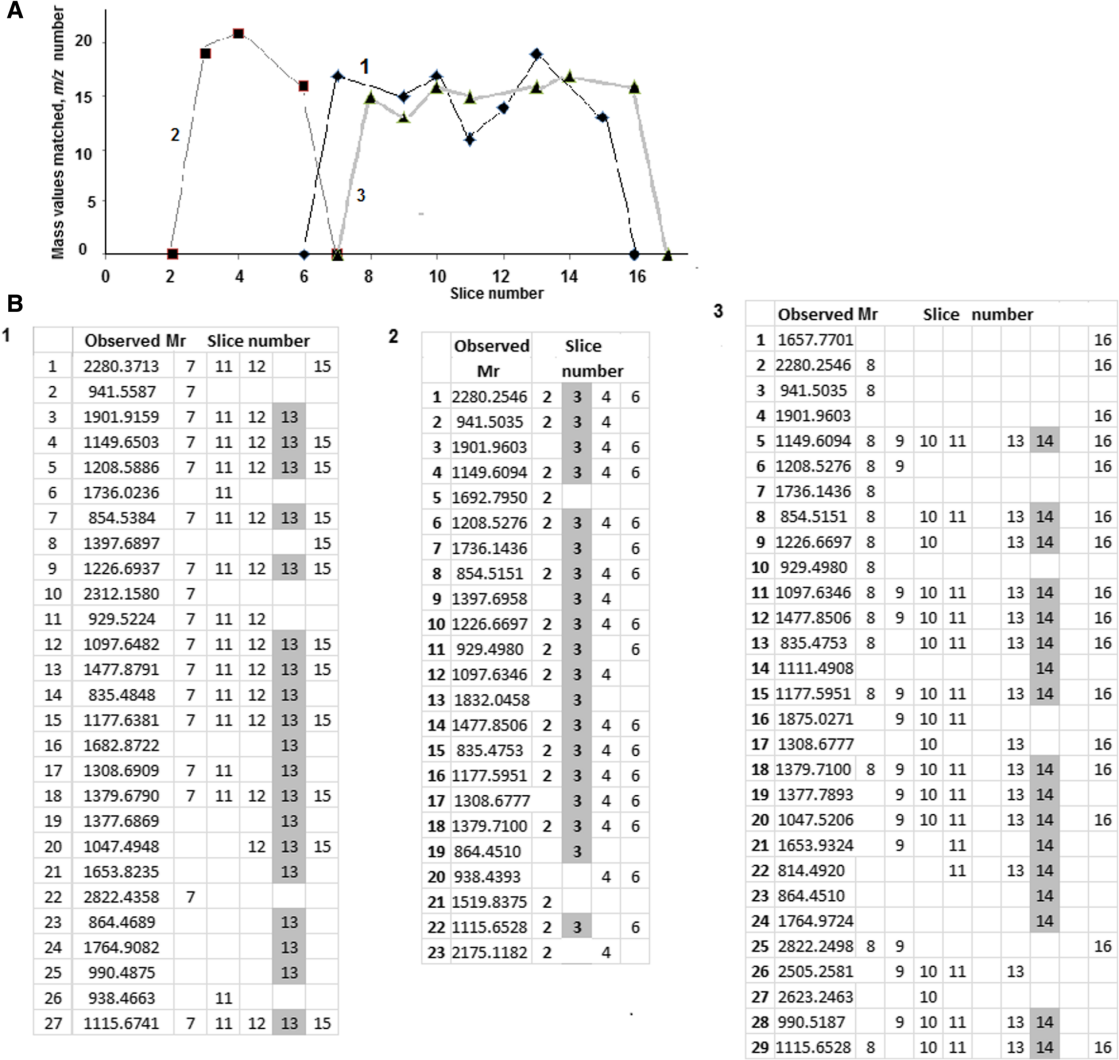
Distribution of the *m/z* values across gel slices **(A)** One-dimensional proteomic profiles of VtgAo2 F1R876 (MW 93213 Da) **(B)** Mapping of peptide masses of VtgAo2 F1R876 identified with PMF onto gel slices (1) Unexposed *Danio rerio* embryos; (2) embryos after exposure to QDs; (3) embryos after exposure to MCS-QDs. The most abundant slices, in which the maximum number of mass values matched the protein, are shown in gray.

When we compared the hydrolysis peaks (*m/z*, peaks in the PMF spectrum) of the Vtgs from embryos exposed to QDs or MCS-QDs with those from the unexposed samples, all the samples had similar numbers of peaks, including the peaks for VtgAo1 F1R887 (MW 148750 Da) and F1Q7L0 (MW 149254 Da). For example, F1R887 was identified in 28 ± 7 peaks from embryos without toxic treatment, and in 22 ± 8 peaks after QD exposure (Table 1). The difference between these values was not significant (p > 0.05; *t* statistic; n = 60). The PMF spectra of VtgAo2 (F1R876) contained 27 and 23 masses in the control (untreated) and QD-treated embryos, respectively, 19 of which were common to both groups. The comparison of these spectra allowed us to identify three masses 1692.7950, 1832.0458, and 1519.8375 in the QD-treated embryos that were absent in the control embryos. Instead of these masses, the mass spectra of VtgAo2 F1R876 in the untreated embryos contained tryptic peptides with observed m/z 1208.589, 1477.879, and 1115.674 (Figure 2B1).

### One-dimensional proteomic profiles of Vtg cleavage products and enrichment of their sequence coverage

The Vtgs identified with PMF are presented as one-dimensional proteomic profiles (Figure 2A). The profiles can be thought of as MS densitograms, in which the density corresponds to the number of PMF peaks matching the particular protein within the MS spectrum of a particular gel slice. Three protein profiles of VtgAo2 Q1MTC4 (MW 93213 Da) are shown in Figure 2A, where each profile corresponds to a separate *D. rerio* sample (see figure legend). As shown in Figure 2(A and B), a single Vtg cleavage product could be detected in several neighboring slices along the SDS-PAGE gel gradient. For example, in the unexposed embryos and in the embryos exposed to MCS-QDs, Q1MTC4 was identified in seven slices in both cases (Figure 2A, lines 1 and 3). However, the exposure of the embryos to QDs reduced the number of slices containing Q1MTC4 to three (Figure 2A, line 2).

Analysis of the MS data from adjacent slices made it possible to increase the sequence coverage of the identified Vtgs (Figure 2B1–3). For example, the peptide coverage of the Vtg-derived structure conjugate Q1MTC4 in the so-called “most abundant” slice [32] (no. 13) was 23% in the control (unexposed) embryos. As can be seen, the gray column in the distribution of the peptide masses of Q1MTC4 (Figure 2B1) corresponding to this slice contained the highest number of peptide masses assigned to Q1MTC4. However, six mass/charge values (2280.3713, 941.5587, 2312.1580, 929.5224, 2822.4358, and 938.4663) were not found in slice no. 13, although they were identified in adjacent slices (nos 7, 11, 12, and 15). As a result, the coverage increased to 36% for the unexposed embryos. A similar situation was observed in the MCS-QD-treated embryos. For example, slice no. 14 lacked the nine masses included in Figure 2B3 corresponding to numbers 1, 2, 4, 6, 16, 17, 25, 26, and 27. The inclusion of these masses from the adjacent slices (nos 8–11, 13, and 16) increased the sequence coverage from 20% (for slice no. 14) to 36% after exposure to MCS-QDs. In contrast, after the QD treatment, combining the MS information from slice no. 3 and adjacent slices (nos 2 and 4) increased the sequence coverage of the multidomain variant Q1MTC4 by only 5%, from 20% in the most abundant slice no. 3 (gray column, Figure 2B2) to 24%.

## Discussion

In this study, the sequential thin slicing of an SDS-PAGE gel and subsequent slice-by-slice MALDI-TOF MS were used to comprehensively analyze the Vtg cleavage products from *D. rerio* embryos. This proteomic methodology has previously been used to distinguish the polymorphic variants of human liver cytochromes P450 [32,33] and to study doxorubicin toxicity in a *D. rerio* embryo model [34].

The choice of *D. rerio* as a model organism was based on a number of features characteristic of this fish species, including its optical transparency, its ready permeability to small molecules directly from the incubation medium, and the high degree of homology between the *D. rerio* and human genomes [37]. The developing *D. rerio* can be used to identify and distinguish different types of toxicity [36]. The Vtg concentration is a standard measure of reproductive function in fish, and is routinely used to evaluate the effects of chemical contaminants [38]. Embryonic Vtg cleavage products can also be used as sensitive and dose-dependent indicators of toxic stress [19].

Over the last two decades, QDs have been used in biomedical imaging, photovoltaic applications, and cancer diagnostics and therapy. Although QDs have great potential utility in cancer cell imaging and biomarker detection, some data suggest that these cadmium-containing nanoparticles are cytotoxic [1,36]. The ability of QDs to efficiently transfer energy to acceptor molecules suggests that they could promote the production of reactive oxygen species *in vivo*, inducing oxidative stress and leading to cell damage. Furthermore, changes have been noted in the expression levels of the proteins that regulate the transcription, translation, splicing, and activities of other proteins. It is noteworthy that the molecular weights of some of the proteins identified with this strategy, which are associated with protein catabolic process, are in the range of 30–70 kDa.

The optical properties of the CdSe_core_/ZnS_shell_ QDs studied are consistent with those reported by other authors using different protocols for nanoparticle synthesis [39-41]. The hydrodynamic size of the QDs is about 9.5 nm, allowing their more rapid and easier renal clearance *in vivo*, which should minimize their toxicity [42].

A series of pilot experiments was conducted to determine the toxicity of QDs in *D. rerio* embryos. We found that the QDs exerted no acute toxicity after QD exposure for 48 h at Cd concentrations up to 185 μM. We detected neither “cadmium-like” responses (such as pericardial or ocular edema) nor “not cadmium-like responses” (tail or yolk sac malformations) [36] under our experimental conditions. Figure 1a shows the normally developing embryos in the QD suspension, MCS-QD-containing medium, and control medium. The teratogenic effects of QDs were only evident after exposure for 7 days (data not shown). Because there was no mortality at a concentration of 2 μM QDs (45 μM Cd) in any of the three replicates, this concentration was selected to profile the toxicity of the QDs.

Vtgs are lipoglycophosphoproteins and their phosphate moieties and glycosylation markedly affect their mobility in SDS-polyacrylamide gels [43], so the molecular mass of the Vtgs estimated from the position of the protein on the gel differs from the theoretical molecular mass (in kDa) predicted from their amino acid sequences. Gündel et al. [19] presented data that implied that Vtgs with molecular weight of 150 kDa were detected in 2DE protein spots in the 15–60 kDa region. In our previous study, we also showed that the treatment of *D. rerio* embryos with doxorubicin caused a significant reduction in the number of Vtgs with theoretical molecular weights around 150 kDa, which were identified in the SDS-PAGE gel slices in the region corresponding to 37–75 kDa [34]. Based on these facts, the SDS-PAGE gel lanes (both for the unexposed *D. rerio* embryos and the embryos exposed to QDs or MCS-QDs) in the region corresponding to molecular weights of 35–75 kDa (determined with protein standards) were selected in the present study.

The exposure of whole *D. rerio* embryos to QDs for 48 h caused no marked changes in the proteins separated by SDS-PAGE relating to those of the unexposed embryos. In the MCS-QD-treated embryos, the intensity of the protein bands in the SDS-PAGE gel lanes was slightly greater than was observed for the unexposed embryos, but the numbers of protein bands were the same.

We used PMF to identify the *D. rerio* embryonic proteins for each mass spectrum, across all the samples, slices, and replicates, using a previously reported strategy for obtaining and processing thin gel slices in selected regions of the SDS-PAGE gel [33]. The resulting slices did not usually correspond to protein bands and contained different amounts of the total protein, which was visible as irregular Coomassie staining within the gel regions (Figure 1b). Therefore, for in-gel digestion, we calculated the amount of trypsin *per* gel slice. This equation was previously established and validated using human liver microsomal proteins [33] and embryonic *D. rerio* proteins after doxorubicin exposure [34].

The region of 35–75 kDa in each gel lane was cut into thin slices. Twenty slices were obtained for each embryo group (the unexposed and the two treated embryo samples) and the proteins identified (Figure 1). The average number of peptide peaks in each mass spectrum was calculated. The differences in the peak numbers were not statistically significant, so the treatment of embryos with nanoparticles or a mixture of the components used for QDs synthesis did not alter the number of peptides observed. This means that, irrespective of the embryo sample, the susceptibility of the proteins to hydrolysis was nearly identical.

In fishes, different types of Vtgs can be processed into many different types of yolk proteins and their variants. When the Vtgs accumulate in the oocyte, they are processed into several fragments (i.e., yolk proteins, YP) and then stored in the egg yolk. A single type of Vtg will almost always be detected as multiple protein bands in the ovary (oocyte) or embryo (yolk sac). *Danio rerio* embryo proteins are poorly separated by SDS-PAGE [44], and we found that each gel slice contained several Vtgs. Overall, 12 Vtg fragments with MWs of 90–150 kDa were successfully identified in the unexposed embryos (Table 1). The majority of identified Vtgs were type I (VtgAo1), and two proteins belonged to VtgAo2. As can be seen from Table 1, the cleavage products were present as multidomain variants in the YP pool [13].

Exposure to QDs or MCS-QDs did not affect the total number of Vtg cleavage products relative to those from the untreated embryos. Some YPs (such as Pv) are difficult or impossible to accurately identify with a Mascot search because they contain repetitive serine residues, most of which are phosphorylated [11,17]. Although Pv was not detected in any of the *D. rerio* embryonic groups (both exposed and unexposed), it was present as conjugates, ranging from full Vtg (LvH–Pv–LvL–β’–CT) to LvH–Pv (Table 1). The differences between the protein MWs presented in Table 1 and the predicted masses of vertebrate Vtgs, including the common subdomains predicted from the conserved cleavage sites [13], may be attributable to the presence of various types of protein modifications during embryonic development and/or QD exposure. VtgC was not observed at all, perhaps because it occurs at very low levels in *D. rerio* or because the short gradient (nearly 9 cm) of the SDS-PAGE gel allowed only poor separation. As can be seen from Table 1, six Vtgs identified as full VtgAo1 had approximately similar MWs (149.3 ± 0.3 kDa). To identify the similarities between the detected VtgAo1 homologues (Q1LWN2, F1Q7L0, E9QFD8, F1R2S5, F1QV15, and F1R2T3; Table 1), we constructed a multiple-sequence alignment of these proteins with the UniProt tool. The average sequence identity was 86.4% (data not shown). The greatest similarity was observed between F1Q7L0 (149254 Da), Q1LWN2 (149140 Da), and E9QFD8 (149258 Da), with 93.2% identity. The second group (94.7% identity) included F1R2S5 (149280 Da) and F1QV15 (149920 Da). The third was formed by a single VtgAo1 with MW 148896 Da (F1R2T3).

In this study, the strategy based on cutting SDS-PAGE gel lanes into thin slices and subsequent slice-by-slice MALDI-TOF PMF identification allowed the mass spectrometric information for each protein to be increased by observing each protein across several (4–16) adjacent slices. The one-dimensional protein profiles provided information about the relative abundance of each of Vtg present in all the groups of *D. rerio* studied.

For example, comparison of the VtgAo2 profiles with MW 93213 Da (F1R876) from the control embryos and QD- and MCS-QD-treated embryos indicated diminished levels of VtgAo2 in *D. rerio* treated with QDs (Figure 2, line 2). Similar profiles could be created for other Vtgs, including Q1MTC4, Q1MTC6, F1R2T3, and F1QV15. For instance, VtgAo1 Q1MTC6 (MW 147084 Da) was detected in 19 slices from unexposed embryos but in eight slices after the QD treatment, demonstrating that the content of this Vtg also decreased after the embryos were exposed to QDs (data not shown). After the embryos were treated with MCS-QDs, VtgAo1 (Q1MTC6) was identified in 18 slices, which was almost equivalent to the number in the control embryos. Moreover, MCS-QD exposure did not affect the distribution *per* slice of almost any Vtg cleavage product.

Vtg has been proposed as a biomarker of exposure to estrogenic chemicals in the environment or waste waters because these chemicals induce Vtg synthesis in male fish [45-47]. However, few studies have tested whether Vtg levels are reduced in response to xenobiotic exposure. Kim et al. [48] demonstrated the down-regulated expression of Vtgs in *Daphnia magna* after exposure to CdSe/ZnSe QDs with a surface coating of 3-mercaptopropionic acid exposure. They reported a dose-dependent reduction in Vtg expression caused by cadmium [49]. iTRAQ-based toxicoproteomic studies in fathead minnows exposed to the anabolic steroid trenbolone showed that the abundance of Vtg Q9W6I2_PIMPR was dose-dependently reduced relative to that in the control embryos [50]. The decline in Vtg content observed in the *D. rerio* embryos in response to QDs in the present study is consistent with the data from other authors [48-50].

The analysis of MALDI-TOF mass spectra showed that they differed between the QD-treated and untreated embryos (Figure 2B and Additional file 2: Figure S2). The comparison of these spectra allowed us to identify three tryptic peptides with the sequences AATL***K***DTEAIWAQFK, EP***K***LVQPVALQLVLER, and FE***K***QVILNGQESK in the QD-treated embryos that were absent in the control embryos. Instead of these peptides, the mass spectra of VtgAo2 F1R876 in the untreated embryos contained tryptic peptides with the sequences DTEAIWAQFK, LVQPVALQLVLER, and QVILNGQESK, respectively. As can be seen, all the peptides of Vtg-derived YP F1R876 in the QD-treated *D. rerio* embryos contained missing cleavage sites in their peptide mass fingerprints at lysine residues (***K***). Recently, a remarkable difference was identified in the MALDI mass spectra generated from drug-treated and control mammalian cells, with the emergence of toxin-related MS peaks [51]. The authors suggested using MALDI mass spectra for the rapid differentiation of apoptotic cells from living or necrotic cells. Nearly 30 matching *m/z* values were identified in the mass spectra of embryos after exposure to MCS-QDs, and 23 masses were identical to the control *m/z* values. As in the QD-treated embryos, the PMF spectra of VtgAo2 F1R876 contained some masses (e.g., 1657.7701, 1111.4908, and so on; Figure 2B3) that were only present in the spectrum after exposure to MCS-QDs.

Therefore, despite the absence of teratological effects and the fact that the number of Vtg cleavage products remained the same after exposure to QDs, the changes in the Vtg abundances after the treatment of *D. rerio* embryos with CdSe_core_/ZnS_shell_ QDs may indicate the toxicity of these nanoparticles.

The widely used strategy of finding candidate proteins associated with a toxic response is based on the spot-to-spot analysis of 2DE gels, followed by the identification of the proteins that are differentially expressed [19,52]. For example, when Gündel et al. [19] analyzed the spots on 2DE gels, they identified the same Vtg cleavage products in both ethanol-exposed (for 48 h) and control *D. rerio* embryos. The toxic effect of ethanol exposure manifested as changes in the intensities of certain protein spots on the 2DE gels after ethanol treatment.

A comparison of our results with those obtained with 2DE protein separation showed that 2DE offered no advantage in separating high-abundance embryo proteins, such as Vtgs. For example, Lucitt et al. [5] identified one protein in several protein spots on a 2DE gel. Ponnudurai et al. [52] identified VtgI (VtgAo1) Q504J4 (MW 36409) in three different spots. Multiple proteins can also be identified in single spots [5]. When analyzing the *D. rerio* embryo proteome at 72 *hpf* with 2DE–liquid chromatography–MS/MS and 2DE–MALDI–TOF/TOF, Lucitt et al. identified 10 proteins with different MWs (22–290 kDa) and different pIs (e.g., 5.49 for fast skeletal myosin heavy chain 3 and 8.96 for the 79-kDa protein) in one spot (no. 1167) [5]. Six different Vtg cleavage products were detected among these proteins. Multiple proteins were identified in numerous spots on the 2DE gel (see Supporting Information in Lucitt et al. [5]).

It is noteworthy that one-dimensional proteomic profiling provided greater sequence coverage of high-molecular-weight Vtgs than 2DE. For example, using 2DE separation, Lucitt et al. [5] found that the sequence coverage for VtgAo1 with MW 149452 was only 13%. The separation of *D. rerio* proteins by SDS-PAGE, cutting the gel lanes into thin slices, and compiling the MS data from neighboring slices increased the sequence coverage of VtgAo2 to 40% in the control embryos. For VtgAo1 with MW 149140 (Q1LWN2), the sequence coverage was 30 ± 3%, whereas collecting the MS data from neighboring slices increased it to 42%. On average, our strategy increased the sequence coverage by 13 ± 7% (mean ± SD, n = 9), depending on the embryo sample. These results are consistent with our earlier data for human liver membrane proteins obtained with MALDI-TOF MS, where the increase in sequence coverage for the microsomal proteins was about 12% [32,33].

As well as detecting Vtg cleavage products, SDS-PAGE thin slicing and MALDI-TOF MS analysis allowed the identification of 25, 35, and 21 other proteins in the control, QDs-treated, and MCS-QDs-treated embryos, respectively. Some of the QD-responsive proteins have functions relating to cell death, including a ubiquitin-domain-containing protein (Q6DG43) and a ubiquitin thioesterase, OTU1 (Q567B1) [53]. For example, slice no. 9 of the QD-treated embryos contained OTU1 (Q6DG43), whereas Q6DG43 was not found among the eight proteins identified in slice no. 9 of the control.

Therefore, changes in the Vtg profile may reflect changes in the entire embryonic proteome in response to exposure to toxic substances, and the Vtg profile alone can be used to estimate the toxicity of different compounds. Our study confirms the view of Gagnaire et al. [54] that SDS-PAGE can determine the presence of aberrant forms of Vtg-like proteins, which can be used as general stress indicators.

## Conclusion

SDS-PAGE is usually the preferred method of reducing the complexity of any biological sample and separating the constituent proteins, including both soluble and insoluble proteins. This study presents a strategy based on cutting SDS-PAGE gel lanes into thin slices, with subsequent slice-by-slice MALDI-TOF PMF identification, to obtain detailed information on the Vtg cleavage products from *D. rerio* embryos. Up to 100 μg of total embryonic protein is used in this technique, which is sufficient for a reproducible analysis of Vtgs, and requires no further purification. Three different types of *D. rerio* embryos were investigated and found to yield unique reproducible MALDI-MS patterns, which distinguished QD-treated embryos from untreated embryos and from embryos exposed to MCS-QDs. We conclude that MALDI-TOF MS Vtg profiles generated in the way described here can be used to estimate nanoparticle toxicity.

## Methods

### QDs and their characteristics

The CdSe_core_/ZnS_shell_ DHLA QDs used for the proteomic analysis of *D. rerio* proteins were produced by the Research Institute for Applied Acoustics (Dubna, Moscow, Russia, http://nanotech-dubna.ru). The QDs were used as a water suspension and analyzed at room temperature. Dynamic light scattering measurements were made with a Nicomp™ 380 ZLC zeta potential and particle size analyzer (Particle Sizing Systems, USA). The absorption spectra were measured with an Agilent/HP 8453 UV–visible spectrophotometer. Fluorescence intensity studies (**λ**_**ex**_ = 312 nm) were performed with a Perkin-Elmer LS-55 fluorescence spectrometer (Maryland, USA). The comparison mixture (MCS-QDs) contained cadmium stearate (45 μM Cd^2+^), zinc acetate (8.1 μM), elemental sulfur (8.1 μM), trioctylphosphine (40.5 μM), and selenium (38.7 μM).

### Experimental organism

Wild-type *Brachydanio* (*Danio*) *rerio* embryos were used in this study. The embryo bioassays were carried out according to the OECD Guideline for testing chemicals (http://www.oecd.org/dataoecd/39/59/36817070.pdf). Adult fish aged 6–12 months were purchased from a local pet shop and used to produce the embryos. The fish (males and females in a 1:2 ratio) were kept in a tank at 26 ± 1°C, under 14 hours of daylight, with water filtration and aeration. A net with a maximum mesh size of 2 mm was put onto the aquarium bottom to prevent the fish from eating their eggs. The embryos were transferred to a Petri dish with reconstituted water 3–4 hours after spawning and analyzed with a Leica EZ 4D stereomicroscope (Germany). The embryos were exposed to aqueous suspensions of QDs or MCS-QDs at 3–5 *hpf* and maintained in these solutions at 26 ± 1°C until 52 *hpf*, at a maximum. The test QD and MCS-QD suspensions were prepared in the wells of 24-well plastic microtiter plates (2 mL of suspension *per* well). The selected embryos were then placed into the wells (1 embryo *per* well). The control embryos were placed into QD- and MCS-QD-free embryo medium (aerated and reconstituted water containing 222 mg/L CaCl_2_, 65 mg/L NaHCO_3_, 60 mg/L MgSO_4_, and 6 mg/L KCl) and grown in the microtiter plates in a separate incubator. We examined 29 unexposed embryos, 34 embryos exposed to QDs, and 29 embryos exposed to MCS-QDs. The QDs were added to the embryos at a concentration of 2 μM (0.045 mM Cd). After exposure, all the embryos were analyzed under a Leica EZ 4D stereomicroscope.

### Dechorionation and homogenization

Before the embryo homogenate was prepared for SDS-PAGE and MALDI-TOF MS, the chorion and yolk sac were removed by chemical digestion with pronase (2 mg/ml) for 15–30 minutes at 37°C, and then suspended in a solution containing 55 mM NaCl, 1.8 mM KCl, and 1.25 mM NaHCO_3_ (50 embryos/100 μL, as described previously) [4]. The samples were suspended in 100 mM K-phosphate buffer (pH 7.4) containing 1 mM EDTA, 1 mM dithiothreitol, and 20% glycerol (v/v) at 50 embryos *per* 62.5 μL of buffer. The embryo homogenate was prepared by ultrasonication of the suspension at 4°C with the program for the ultrasonic BANDELIN Sonopuls HD 2070 instrument: 2 cycles of 50 s with a 5 s interval (i.e., 20 active seconds). The ultrasonicated homogenate was centrifuged at 3000 rpm for 40 s using a Hettich Mikro 12-24 centrifuge and the supernatant was used for SDS-PAGE [34].

### SDS-PAGE, gel slicing, and in-gel digestion

The Mini-Protean® III System (Bio-Rad Laboratories, Russian Federation) was used for the SDS-PAGE separation of the *D. rerio* embryo proteins, according to the manufacturer’s instructions. The supernatant of the homogenate (above) was mixed with three volumes of sample buffer (0.5 M Tris-HCl [pH 6.8], 2% SDS, 10% glycerol, 5% 2-mercaptoethanol, 0.5% bromophenol blue) and incubated at 95°C for 4 min. Electrophoretic separation was performed in 12.5% polyacrylamide gels (9 cm) using the method of Laemmli [55]. Equal amounts of protein (an aliquot of ultrasonicated homogenate containing four embryos) from unexposed embryos or embryo that had been exposed to QDs or MCS-QDs were loaded into three parallel gel lanes. After the proteins were separated according to their mass, the gels were stained with 0.1% Coomassie Brilliant Blue G-250, as described previously [40]. The gel were scanned and a densitometric analysis was performed with the Quantity One® software (version 4.6.1) (Bio-Rad, UK). After separation, a 10 mm region of each gel lane corresponding to molecular weights of 37–75 kDa (a prestained molecular mass marker was used to estimate the position of this region) was cut sequentially into thin slices (about 0.2 mm), as described by Petushkova et al. [33]. In brief, each of the 20 slices was divided into three equal fragments (piece sizes, 0.5 × 0.8 × 2 mm^3^). The middle piece of each slice was sampled, washed three times with water to remove the dye, and incubated in destaining buffer (50% acetonitrile [v/v] in 100 mM ammonium bicarbonate [pH 8.9]) for 20 min at 56°C. After dehydration with 100% acetonitrile for 20 min, the slices were subjected to in-gel proteolysis with trypsin [56]. For this purpose, 6.3 ± 2.0 μL of trypsin solution (25 ng/μL modified trypsin in 50 mM bicarbonate ammonium) was added to each piece of gel, depending on its relative staining [33], and the mixture was incubated overnight at 37°C. Then 15 μl of 0.7% trifluoroacetic acid was added to each gel piece and the samples were incubated for 2 h at room temperature. The mixture of proteolytic peptides from the gel piece was used for MS analysis.

### MALDI-TOF PMF

The extracted tryptic peptides obtained from the slice fragments (1 μL) were spotted onto a MALDI target (MTP 600/384 Anchor Chip™; Bruker Daltonics, Germany), mixed with 1 μl of matrix solution (20 mg/ml 2,5-dehydrobenzoic acid in 20% acetonitrile and 0.1% trifluoroacetic acid), and air dried. Three replicates of each sample were placed on the MALDI target. The MS analysis was performed in the reflection/delayed extraction mode at an accelerating voltage of 25 kV with a 135 ns delay, using an Ultraflex II mass spectrometer (Bruker Daltonics). Typically, each mass spectrum was the sum of 100 laser shots. From each target spot, 4–6 mass spectra were acquired. Laser fluency was adjusted to above the desorption threshold of the matrix to obtain the best resolution and the most accurate mass measurements. Signals with a signal/noise ratio > 6 and a maximum of 69 ± 12 peaks *per* spectrum were used to build peak lists with the SNAP algorithm (FlexAnalysis 2.0, Bruker Daltonics), and the resulting mass spectra were calibrated with trypsin autolysis products (*m/z* 842.5094 Da and 2211.1046 Da).

### Data interpretation

The resulting peak lists were used to search the UniProtKB/Swiss-Prot database (ftp://ftp.ebi.ac.uk/pub/databases/uniprot/current_release/knowledgebase/complete/). Identification by PMF was performed with the Mascot search engine and the following parameters: (1) trypsin cleavage, allowing up to one missed cleavage; (2) variable modification, including propionamide (C), oxidation (M) and oxidation (HW); (3) mass tolerance, 90 ppm; and (4) the Danre_proteome as the source database for protein identification. Protein scores greater than 59 were considered significant.

The data were analyzed statistically with Student’s *t* test in Microsoft Excel 2010. The differences between means were considered significant at p < 0.05.

## Additional files

Additional file 1: Figure S1. Assessment of the CdSe/ZnS–DHLA QD sizes and their optical properties. a) A schematic image; b) Dynamic light scattering measurements of the distribution of particle sizes. To determine the size distribution, a concentrated nanoparticle suspension was diluted with water (0.1 mM) and analyzed at room temperature: *d* ~9.5 nm, 0.045 mM Cd^2+^. C) Absorption spectrum in H_2_O; d) Fluorescence emission spectrum in H_2_O (λ_ex_ 312 nm). DHLA (dihydrolipoic acid). Additional file 2: Figure S2. VtgAo2 F1R876 identified with MALDI-TOF MS differed in the SDS-PAGE slices from QD-treated and untreated *D. rerio* embryos. Labeled peaks (*) were only observed in the mass spectra of the treated embryos.

## Abbreviations

QDs: Quantum dots
DHLA: Dihydrolipoic acid
Vtg: Vitellogenin
Vtgs: Vitellogenin cleavage products
SDS-PAGE: Sodium dodecyl sulfate polyacrylamide gel electrophoresis
MALDI-TOF: Matrix-assisted laser desorption/ionization
MS: Mass spectrometry
PMF: Peptide mass fingerprinting
*hpf*: Hours post-fertilization
*dpf*: Days post-fertilization
1D-proteomic profile: One-dimensional proteomic profile
2DE: Two-dimensional electrophoresis
MCS-QDs: A mixture comprising components for quantum dots synthesis
ISO: International Organization for Standardization
OECD: Organization for Economic Co-operation and Development
US EPA: United States Environmental Protection Agency
YP: Yolk protein
LvH: A heavy chain lipovitellin
Pv: A phosvitin
LvL: A light chain lipovitellin
β: The beta component
CT: C-terminal coding region
iTRAQ: isobaric tags for relative and absolute quantification
