# Supplementary figures and images for: One-dimensional proteomic profiling of Danio rerio embryo vitellogenin to estimate quantum dot toxicity
Source: Proteome Sci. 2015 May 2;13:17. doi: 10.1186/s12953-015-0072-7 (PMC4426544; doi:10.1186/s12953-015-0072-7)

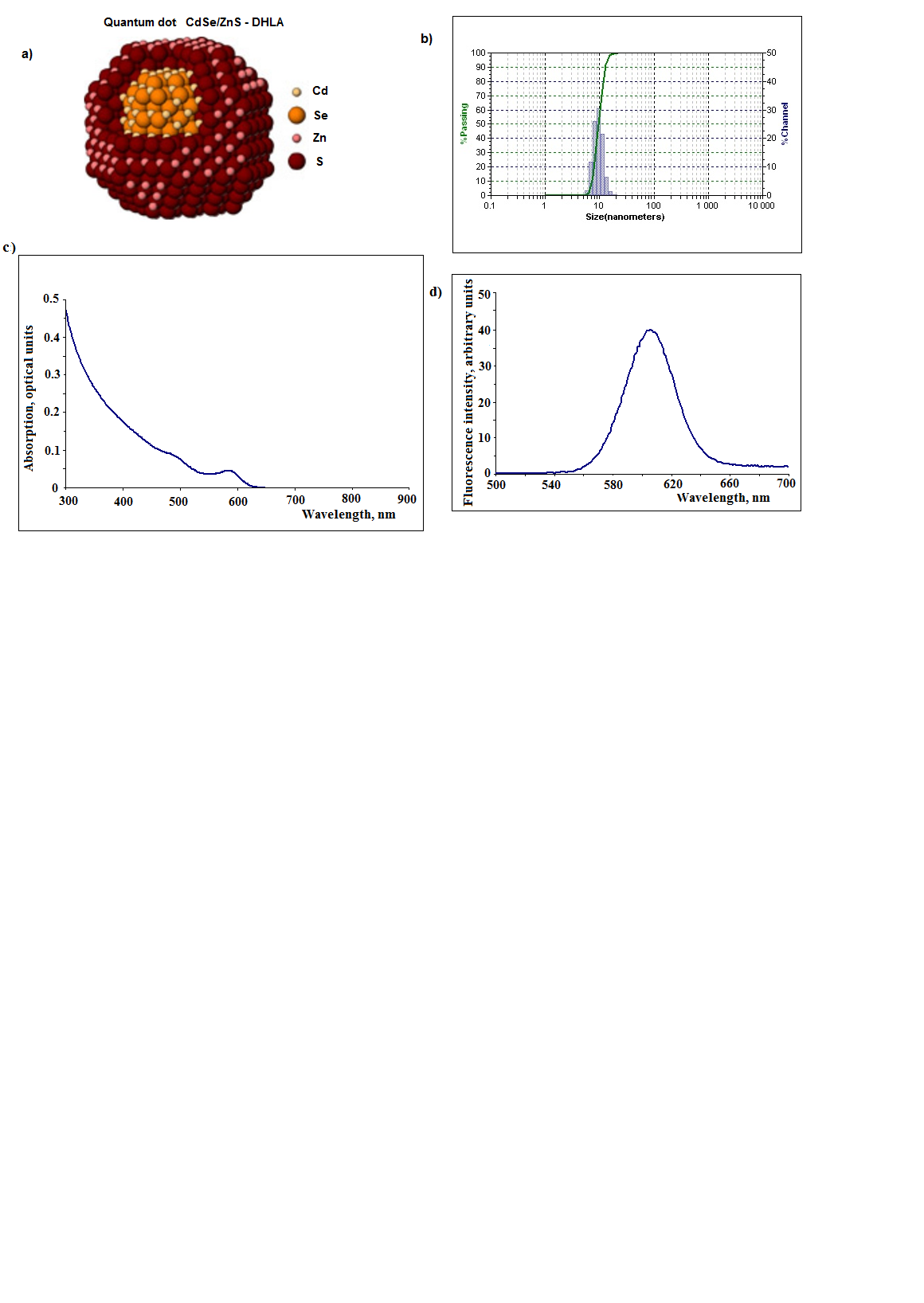

Supplement: Additional file 1: Figure S1. — Assessment of the CdSe/ZnS–DHLA QD sizes and their optical properties. a) A schematic image; b) Dynamic light scattering measurements of the distribution of particle sizes. To determine the size distribution, a concentrated nanoparticle suspension was diluted with water (0.1 mM) and analyzed at room temperature: d ~9.5 nm, 0.045 mM Cd2+. C) Absorption spectrum in H2O; d) Fluorescence emission spectrum in H2O (λex 312 nm). DHLA (dihydrolipoic acid). [file 12953_2015_72_MOESM1_ESM.tiff]

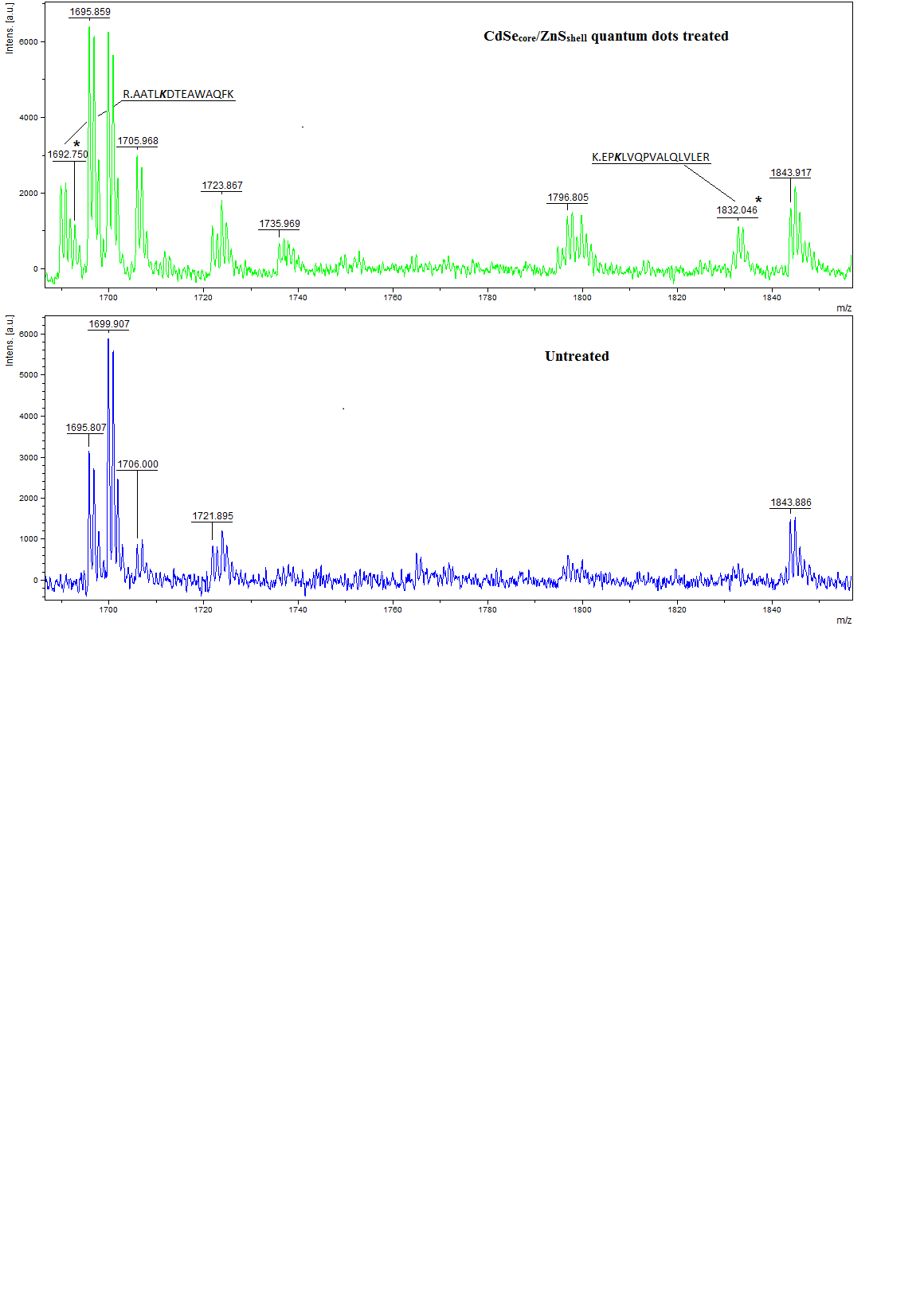

Supplement: Additional file 2: Figure S2. — VtgAo2 F1R876 identified with MALDI-TOF MS differed in the SDS-PAGE slices from QD-treated and untreated D. rerio embryos. Labeled peaks (*) were only observed in the mass spectra of the treated embryos. [file 12953_2015_72_MOESM2_ESM.tiff]
